# Adaptive metabolic rewiring to chronic SFK inhibition

**DOI:** 10.18632/oncotarget.8146

**Published:** 2016-03-17

**Authors:** Edgar Pinedo-Carpio, David Davidson, Veronica L. Martinez Marignac, Justin Panasci, Raquel Aloyz

**Affiliations:** ^1^ Jewish General Hospital, Lady Davis Institute & McGill University, Faculty of Medicine, Division of Experimental Medicine & Department of Oncology, Montréal, Québec H3T 1E2, Canada; ^2^ CICYTTP IBIOGEM, CONICET, Diamante Argentina, Diamante CP3105, Entre Rios Argentina

**Keywords:** acquired resistance, SKF inhibition, PI3K, TGFβ, metabolism

## Abstract

Src family kinases (SFK) are key regulators of cellular proliferation, differentiation, survival, motility and angiogenesis. As such, SFK inhibitors are being tested in clinical trials to prevent metastasis as an alternative to current treatment regimens for a variety of cancers including breast cancer. To contribute to the development of molecular tools improving SFK-targeted therapies, we used the SFK inhibitor dasatinib and a well characterized triple negative breast cancer cell line (BT20). Comparison of the response of BT20 cells with acquired resistance to dasatinib and its’ parental counterpart suggest that chronic exposure to SFK inhibition results in increased dependency on TGFβ signaling for proliferation, both in the absence or the presence of dasatinib. In addition, we found that acquired (but not de novo) resistance to dasatinib was reduced by non-cytotoxic concentrations compounds hindering on PI3K, mTORC1 signaling, endoplasmic reticulum stress or autophagy.

## INTRODUCTION

Small molecule inhibitors targeting the Src family kinases (SFK)s are currently in clinical trials to test their efficacy in the treatment of metastatic breast cancer (NCT01471106) [1]. SFKs are known oncogenes that function in the regulation of cellular proliferation, adhesion, motility and epithelial to mesenchymal transition. As such, inhibiting SFKs may have therapeutic benefit for cancer patients [2, 3]. Phenotypic aberrations in the cellular processes mentioned above are often associated as well with non-canonical TGF-β signaling [4–6]. Indeed, as reported for SFK inhibitors, TGF-β inhibitors can reduce bone metastasis of breast cancer and melanoma [7–9]. Problematically, the therapeutic use of TGF-β inhibitors has been hampered by the contrasting dual roles of TGF-β signaling in cancer cell proliferation (i.e. inhibits the growth of early stage tumors and promotes the growth of late stage tumors). In the present work, we report that acquired (but not *de novo*) resistance to dasatinib is mediated by targetable changes in signaling pathways previously reported to mediate drug resistance in triple negative breast cancer (TNBC) including TGF-β signaling, PI3K/mTORC1, endoplasmic reticulum stress and autophagy [10–13]. Noteworthy, in primary leukemic lymphocytes dasatinib resistance was shown to be associated with differential dependency on autophagy, mTORC1 and AMPK signaling for survival [14]. The involvement of AMPK, ER-stress and autophagy in dasatinib resistance have been recently reported as well in prostate cancer cells [5, 6]. AMPK is a sensor of bioenergetic stress activated by LKB. Generally speaking AMPK promotes catabolic process to generate ATP while inhibiting anabolic process to save energy [15]. As AMPK acts as a bioenergetic rheostat, another kinase, mTORC1 acts as a rheostat of nutrient availability (i.e. amino acids, glucose and oxygen)[16]. In other words when nutrients are limiting mTORC1 signaling is attenuated but increased in nutrient reach conditions promotes anabolism and cell growth. mTORC1 is activated downstream the PI3K/AKT axis and promotes protein synthesis initiation by phosphorylating S6Ks and 4EBPs [17] and inhibiting catabolic processes such as autophagy [18]. Autophagy is a catabolic process that cells can use to generate energy by the oxidation of cellular components and is promoted by AMPK and inhibited by mTORC1 [19]. Autophagy can also be triggered by reactive oxygen species (ROS) and endoplasmic reticulum stress [20]. ER-stress, namely, can also confer resistance to AMPK activator metformin, and specifically, after ER-stress, cancer cells can survive through autophagy [21]. Autophagy was previously shown to facilitate acquisition of a CD44+24- breast cancer stem cell phenotype, effect that was prevented by chloroquine (an inhibitor of autophagy) [22].

Here we report that in a well characterized TNBC cell line, BT20, sustained exposure to dasatinib results in the selection of tolerant cells to the drug, increased dependency on pharmacologically targetable signaling pathways, namely PI3K, autophagy and TGFβ.

## RESULTS

### SFK-independent PI3K/mTORC1 signaling sustains proliferation of dasatinib resistant BT20 cells

**Figure 1:**
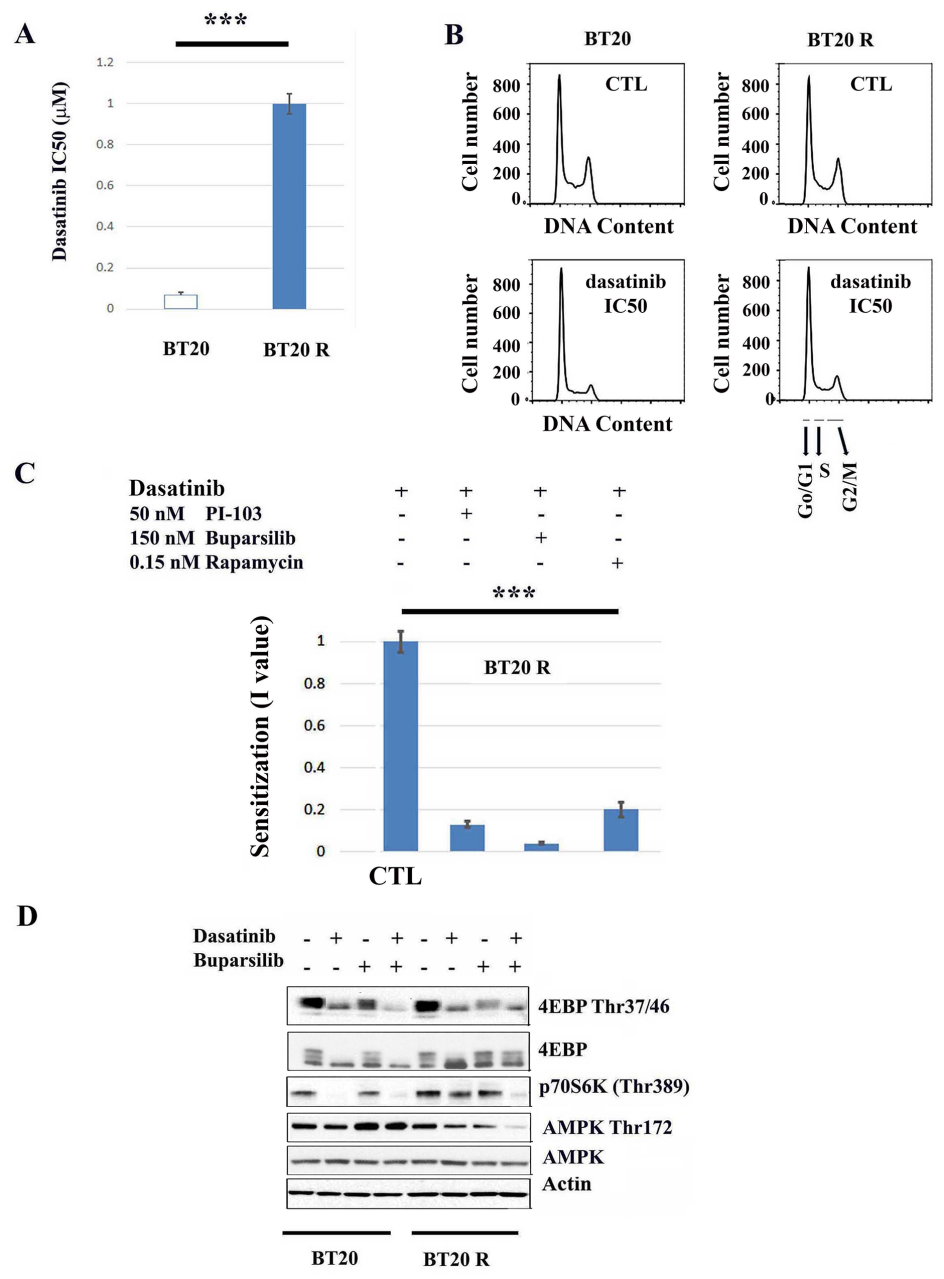
Inhibitors of the PI3K/mTORC11 pathway enhance the antiproliferative effect of dasatinib in resistant BT20 cells **A**. Exponentially growing parental (BT20) and resistant (BT20R) cells were exposed to dasatinib (0-5 μM) for 5 days and the IC_50_ concentrations (indicated in the y-axis) obtained using the SRB assay. The bars represent the mean value of 5 independent experiments plus minus the standard error. The asterisks (***) indicate a p<0.001 (paired t-test). **B**. Exponentially growing parental (BT20) and resistant (BT20R) cells were treated with vehicle or the respective dasatinib IC_50_ concentration (0.04 and 1 μM respectively) for twenty four hours and changes in cell cycle progression as described. The y-axis indicates cell number and the x-axis DNA content. As indicated by the arrows differences in DNA content correspond to the different cell cycle phases: Go/G1 (2N), S (>2N), G2/M (4N). Representative results of 3 experiments. **C**. The graph bars represents the mean I values (n=4) plus/minus the standard error, obtained as described in materials and methods using the SRB assay 5 days after treatment. BT20R cells were treated with dasatinib (0-5 μM) in combination with vehicle (CTL), PI3-103 (50nM), Buparsilib (150 nM) or Rapamycin (0.15 nM). Statistical analysis indicates that the sensitization I values obtained by the combination of dasatinib with the indicated inhibitors are lower than the values obtained by the combination of dasatinib with vehicle. The asterisks (***) indicate a p<0.001 (One way ANOVA), followed by t-test with p<0.05. **D**. The effect of dasatinib (respective _IC50_ concentrations) alone or in combination with 150 nM Buparsilib on the phosphorylation state of mTORC1 downstream targets (4E-BP1 and p70S6K) or AMPK was assessed by western blot analysis. BT20 or BT20R cells were treated for forty-eight hours as indicated and phosphorylation of the indicated targets was assessed in 50μg of proteins extracts. Equal loading was confirmed by re probing with an α-actin antibody.

Chronic exposure to non-toxic concentrations of dasatinib resulted in a 25 fold increase resistance to the drug (i.e. change in the IC_50_ concentration from 0.04 to 1 μM) (Figuare 1A and Supplementary Figure S1). Treatment with the respective IC_50_ concentrations of dasatinib resulted in G0/G1 cell cycle arrest in both BT20 parental cells (parental hereafter) and BT20R cells with acquired resistance to the drug (resistant hereafter) (Figure 1B). Acquired resistance was reversible, since resistance to dasatinib was lost after 15 passages in the absence of selective pressure suggesting that acquired resistance to dasatinib in BT20 cells is mediated by a non-mutational mechanism [23]. We assessed the effects of inhibitors of PI3K and mTORC1 on the sensitivity to dasatinib using non-toxic concentrations of two PI3K inhibitors, PI-103 (50 nM) or Buparsilib (150 nM) and the mTORC1 inhibitor rapamycin (0.15 nM) [24–26]. With no difference between cell lines in the sensitivity to these drugs when used alone (data not shown), in contrast, PI-103, Buparsilib or rapamycin significantly sensitized resistant cells (BT20R cells) to dasatinib (by 7.5, 25 and 5 fold respectively) (Figure 1C). These drugs did not affect dasatinib resistance in BT20 parental cells (Supplementary Figure S2). Consistent with an inhibitory effect of dasatinib on mTORC1 activity, treatment with the drug decreased both phospho-4E-BP1 Thr37/47 and p70S6K Thr398 in parental cells. In contrast, upon treatment of resistant cells with dasatinib, abrogation of phospho-p70S6K Thr398 was achieved by the combination of dasatinib and 150 nM buparsilib but not by dasatinib alone (Figure 1D). Additionally, even though we did not find differences in basal AMPK phosphorylation between parental and resistant cells, buparsilib showed opposing effects on AMPK Thr172 in parental and resistant cells. A non-toxic concentration of Buparsilib increased AMPK Thr172 in sensitive BT20 cells while decreasing AMPK Thr172 in resistant BT20 cells. This suggests that PI3K signaling promotes metabolic homeostasis in sensitive but not resistant BT20 cells [15]. Although dasatinib did not affect AMPK phosphorylation status, the drug exacerbated the effect of Buparsilib in resistant BT20 cells (i.e. decreased AMPK Thr172) (Figure 1D). The combination of dasatinib with PI3-103, Buparsilib or Rapamycin did not affect the expression of the apoptotic marker Annexin V (data not shown). Suggesting enhancement by the drugs of the cytostatic effect of dasatinib. Importantly, the concentrations of PI3-103, Buparsilib or Rapamycin used in combination with dasatinib were not cytotoxic or cytostatic when used alone.

### ER stress and autophagy contribute to acquired resistance to dasatinib in resistant BT20 cells

**Figure 2:**
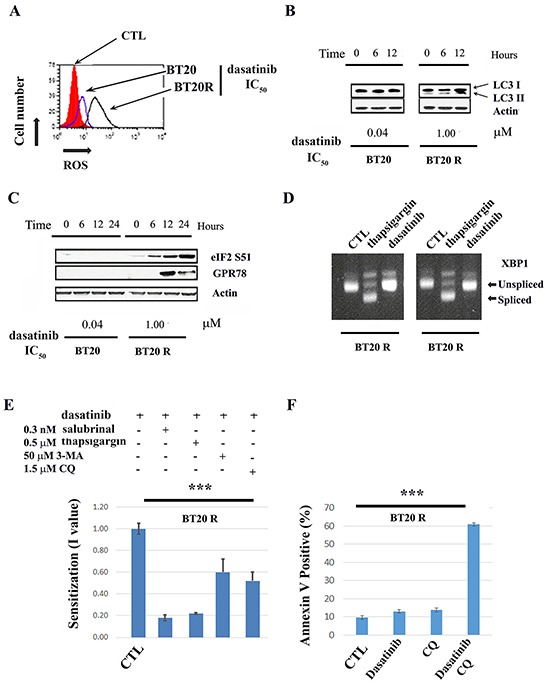
Drug-induced ER-stress and autophagy contribute to acquired resistance to dasatinib **A**. Changes in the redox state of parental (BT20 blue line) or resistant (BT20R black line) cells after twelve hours after dasatinib treatment with the (respective IC_50_) is shown with respect to vehicle treated cells (in red). A representative experiment of three is shown. **B**. Conversion of LC3-1 to LC3-2 in parental BT20 or resistant BT20R cells was assessed by western blot analysis 0 to 6 hours after treatment with dasatinib IC_50_. Equal protein loading (50μg) was confirmed by re probing with an α-actin antibody. **C**. Changes in GPR78 expression levels and eIF2 phosphorylation in parental BT20 or resistant BT20R cells was assessed by western blot analysis 0 to 6 hours after treatment with dasatinib (respective IC_50_). Equal protein loading (50μg) was confirmed by re probing with an α-actin antibody. **D**. XBP-1 splicing was assessed in BT20 and BT20R cells twelve hours after treatment with vehicle (CTL), thapsigargin (THAP, 500 nM positive control) or dasatinib treatment (DAS, IC_50_ concentration). The arrows indicate XBP-1 splice variants. **E**. BT20R cells were treated with dasatinib (0-50μM) in combination with vehicle, non-toxic concentrations of drugs affecting the ER stress response (i.e. the inhibitor salubrinal or the inducer thapsigargin) or inhibitors of early (3-MA) or later (CQ) autophagy as indicated. Five days after treatment dasatinib IC_50_s in the combinations were obtained utilizing the SRB assay and used to calculate the sensitization values (I value) for each combination. The y-axis represent the mean I values (n=5) plus minus the standard error. The *** indicate a significant difference between the I values with respect to 1 (i.e. dasatinib in combination with vehicle) using ANOVA with p<0.001 followed by t-test. **F**. BT20 R cells were treated with vehicle (CTL), CQ (1.5 μM), dasatinib IC_50_ (1μM) or the combination of CQ and dasatinib for seventy two hours. Annexin V expression was assessed by flow cytometry as described in materials and methods. Increase in cell death by the combination of dasatinib plus CQ respect to the drugs alone was assessed by one way ANOVA (***p<0.001). The bars graph represent the mean values of Annexin V positive cells plus minus the standard error (n=4).

Consistent with differences in metabolic rewiring between BT20 sensitive and resistant cells, dasatinib induced the production of reactive oxygen species (ROS) (Figure 2A) and cleavage of LCI (a marker of autophagy) (Figure 2B) preferentially in resistant cells. In addition, following the same pattern, dasatinib-induced elongation factor eIF2α (eIF2α) phosphorylation and increased levels of the chaperone, 78kDa glucose-regulated protein (GRP78), two hallmarks of ER stress induction [27–29] (Figure 2C). To further analyze the effect of dasatinib on the ER-stress/unfolded protein response, we analyzed for XPB1 alternative splicing after dasatinib treatment (IC_50_ concentration). The results we obtained suggest that induction of ER stress in resistant cells (BT20R) by dasatinib is not associated with induction of cell death since we were not able to detect the XPB1 splice variant required for transcription of death effectors (Figure 2D). Indeed, an inhibitor of the ER stress response (salubrinal (0.5 μM) increased dasatinib sensitivity by 5 fold in BT20R cells. We also observed a sensitization effect by co-treatment with an inducer of ER-stress, thapsigargin (0.2 nM). Together, these observations suggest regulation of the ER-stress response is important for maintenance of the survival of resistant cells under dasatinib-induced stress. Co-treatment with inhibitors of early or late autophagy using 3-MA or Chloroquine (CQ) respectively, resulted in a more discrete but significant (2 fold) sensitization to dasatinib as well (Figure 2E). In contrast, these drugs (i.e. salubrinal, thapsigargin, 3-MA or CQ) did not affect dasatinib resistance in BT20 parental cells. Unlike the results we obtained with inhibitors of PI3K or mTORC1, pharmacological interference with ER stress or autophagy resulted in increased cell death in BT20R cells. Representative result using Annexin V induction by dasatinib, CQ or their combination are shown in Figure 2F. Our results suggest that in BT20R cells tight regulation of the ER stress response is required to maintain homeostasis and survival under dasatinib-induced stress. Importantly, the concentrations of 3-MA, CQ, salubrinal or thapsigargin used were not cytotoxic (nor cytostatic) when used alone.

### TGFβ signaling contributes to dasatinib acquired resistance in BT20 cells

**Figure 3:**
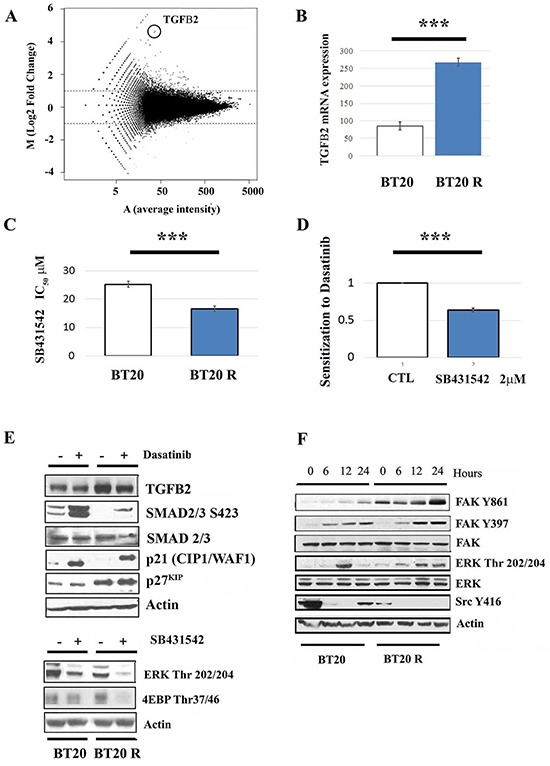
signaling contributes to acquired resistance to dasatinib **A**. Volcano analysis showing differential gene expression in BT20 cells with acquired resistant to dasatinib (upper panel). **B**. Overexpression of 2 was confirmed by RT-PCR as described in materials and methods. *** indicates that BT20R cells express significantly higher levels of mRNA coding for TGFβ2 respect to parental BT20 cells with p<0.001 obtained after paired t-test analysis. The bars represent the mean value of 3 independent experiments plus/minus de standard error. **C**. Sensitivity to TGFβR inhibition was assessed using the SRB assay. The bar graph represents the mean SB43542 IC_50_ value (n=3) in parental (BT20) and resistant cells (BT20R) plus minus the standard error (upper panel). The asterisks (***) indicate a p<0.001 (t-test). **D**. BT20R cells were treated with dasatinib (0-50μM) in combination with vehicle or a non-toxic concentrations of SB43542 (2μM). Five days after treatment dasatinib IC_50_s in the combinations were obtained utilizing the SRB assay and used to calculate the sensitization values (I value). The y-axis represent the mean I values (n=3) plus/minus the standard error. The *** indicate a significant differences in I values with respect to the CTL (i.e. dasatinib + vehicle) using paired t-test with p<0.001. **E**. Changes in 2, p21^WAF^, p27^KIP^ expression levels and phosphorylation status of SMAD2/3 was assessed by western blot assay twelve hours after treatment with vehicle or the IC_50_ concentration of dasatinib (upper panel). Treatment with the respective SB43542 IC_50_ concentration for 12 hours resulted in decreased ERK and 4E-BP1 phosphorylation (lower panel). Equal protein loading (50μg) was assessed by re probing with an α-actin antibody. Representative experiment of three. **F**. The effect of dasatinib on FAK, ERK and Src phosphorylation status was assessed by western blot analysis, 0 to 24 hours after treatment with dasatinib IC_50_ concentration. Equal protein loading (50μg) was assessed by re probing with an α-actin antibody. Representative experiment of three.

Differential gene expression between parental and resistant cells was analyzed using RNA-Seq as previously described [30]. Analysis of 157,601 transcripts from Ensembl Hg19 revealed that 224 transcripts (including isoforms) were differentially expressed in acquired resistant BT20 cells with respect to parental cells (adjusted FDR p-value < 0.1 and at least 2-fold expression change). A Volcano plot displaying differential expression is shown in Figure 3A. Ninety genes were differentially expressed, seventy three upregulated with four targeted candidate genes (FOXO4, IDH2, TGFβ2 and FN1) and seventeen downregulated genes (Supplementary Data Gene Expression). Differential expression of TGFβ2 was confirmed by qPCR (Figure 3B). Changes in expression of these genes was also shown to be significant using standard mRNA expression profiling (Affymetrix platform HU133 oligonucleotide array) [31] (data not shown). Differentially expressed gene function was estimated using Gene Ontology (GO) annotation. A total of 116 differentially expressed genes were assigned to GO terms in biological processes category. These genes function in multicellular organismal development (23), multicellular organismal process (29), developmental process (26), multicellular organismal development and system development (18) and anatomical structure development (19) (Supplementary Data Gene Expression).

### Canonical TGFβ signaling is down regulated in BT20 cells with acquired resistance to dasatinib

To determine the role of TGFβ signaling, we utilized the TGFβI receptor inhibitor SB431542 [32]. A shift in TGFβ signaling is suggested by a two fold increase in SB431542 IC_50_ concentration in resistant respect to sensitive BT20 cell (Figure 3C). In addition, only in BT20 resistant cells, a non-toxic concentration of SB431542 (2 μM) significantly decreased dasatinib IC_50_ by half (Figure 3D). Suggesting TGFβ signaling promotes survival in resistant BT20 cells treated with dasatinib. Similar results were obtained using the TGFβRI receptor inhibitor LY364947 (data not shown). Autocrine TGFβRI signaling in BT20 cells is suggested by the presence of SMAD2/3 basal phosphorylation in untreated parental BT20 cells. Decreased basal SMAD2/3 phosphorylation in resistant cells suggests that chronic exposure to dasatinib results in decreased canonical-TGFβ signaling. In both parental and resistant cells dasatinib promoted canonical TGFβ signaling, preferentially in the parental cells with enhanced SMAD2/3 phosphorylation, p21^Cip^ and p27^WAF^, suggesting that this TKI promotes (or de represses) canonical TGFβ signaling preferentially in parental BT20 cells (Figure 3E upper panel). In contrast, in resistant BT20 cells, with increased TGFβ2 both at the mRNA and protein levels, dasatinib treatment had a mild effect on SMAD2/3 phosphorylation but induced sustained ERK and FAK Y861 phosphorylation. The contribution of TGFβ signaling to ERK and mTORC1 activation in resistant cells is suggested by the inhibitory effect of SB431542 on ERK and 4E-BP1 phosphorylation (Figure 3E lower panel). These results together with dasatinib induced decreased TFGβ2 levels in both parental and resistant cells, suggesting that the dasatinib promotes autocrine TGFβ signaling (Figure 3F) with a shift towards non-canonical TGFβ signaling in BT20 resistant cells.

## DISCUSSION

Hypersensitivity to SFK inhibitors with reduced development of metastasis in a variety of cancers including TNBC cells has been reported. SFKs contribute to full activation of RAS and PI3K by growth factors, cytokines and integrins. In breast cancer, SFKs can activate or modulate mTORC1 signaling by AKT dependent and independent mechanisms [16, 33–39]. For example by regulating S6K activity and localization and by preventing rapamycin-induced feedback activation of AKT [40]. Conversely, inhibitors of the PI3K/mTORC1 pathway have been reported to sensitize human lung cancer cells to dasatinib [41, 42]. Our results suggest a differential role of SFKs in the regulation of the PI3K/mTORC1 signaling axis in cells chronically exposed to dasatinib.

As previously reported, in parental BT20 cells activation of the PI3K/mTORC1 pathway seems to be dependent on SFK activity, since dasatinib abrogated both 4E-BP1 and p70S6K [43–45]. In contrast, in dasatinib resistant BT20 cells, dasatinib failed to decrease p70S6K phosphorylation, effect that was achieved by co-treatment with buparsilib. Since buparsilib sensitized resistant cells to dasatinib, it is possible that SFK-independent activation of the PI3K/mTORC1 axis contributes to acquire resistance to dasatinib in BT20 cells. This is in agreement with previous reports that suggest a complementary role between SFKs and PI3K signaling [46].

TGFβ signaling is a commonly deregulated pathway in breast cancer promoting proliferation and survival by a shift towards non-canonical TGFβ signaling. Suggestive of autocrine non-canonical TGFβ signaling in BT20 cells is the reduction on 4E-BP1 and ERK phosphorylation following SB431542 treatment in both sensitive and resistant BT20 cells. Chronic exposure to dasatinib resulted in increased expression of TGFβ2 (both at the mRNA and protein levels) and increased resistance to a TGFβ inhibitor. This increase in TGFβ signaling occurred with a shift towards non-canonical TGFβ signaling as suggested by decreased SMAD2/3 S423 in resistant cells. These results taken together suggest that BT20 cells can use non-canonical TGFβ signaling to promote proliferation under chronic inhibition of SFKs [47–49]. This is supported by the synergistic effect of SB431542 when used in combination with dasatinib in BT20 resistant cells.

Regulation of cell cycle by SFKs has been recently suggested in a variety of cancers including breast carcinomas where Src phosphorylation inversely correlated with p27 KIP expression [50]. Dasatinib-induced p21^WAF^/and/or p27^KIP^ levels has been previously reported in leukemic and lung cancer cells [38, 41, 50, 51]. Noteworthy, BT20 resistant cells expressed high basal p27 ^KIP^ levels when compared to parental cells. Interestingly, it is possible that p21^WAF^ plays an oncogenic role in BT20R cells. This is suggested by a shift on the molecular weight of this cyclin-dependent kinase inhibitor (often associated with phosphorylation) that was evident in BT20R but not in BT20 parental cells [52]. Together with differences in PI3K and TGFβ signaling our results demonstrate that chronic exposure to dasatinib affects metabolic rewiring in BT20 cells. Maintenance of survival upon cell cycle arrest in dasatinib treated-resistant cells was dependent on ER-stress signaling and autophagy, suggesting a metabolic shift in BT20 cells chronically exposed to the TKI. For instance, the mTOR-p70S6K axis has been shown to suppress mitochondrial respiration in BT20 cells which are highly dependent on glycolytic metabolism [53]. Dasatinib has been shown to affect glucose metabolism in primary leukemic cells and in CML patients treated with the drug [14, 54]. Induction of ER stress and autophagy by dasatinib has been reported in the literature in human cancer cells [14, 55, 56]. Both processes (autophagy and ER stress) are tightly regulated processes that can contribute to survival and drug resistance in cancer cells [19, 36, 57–63] [64–69]. Autophagy and ER-stress can promote survival by promoting the recycling of cellular components, including damaged organelles and by contributing to inhibition of mRNA translation [70–72]. This has been shown in in breast cancer cells with acquired resistance to the AMPK activator metformin, where autophagy mediates survival upon ER stress induction [21]. All together our results suggest that chronic treatment with dasatinib might result in the selection of resistant cells with pharmacologically targetable metabolic rewiring contributing to drug resistance including SFK-independent activation of PI3K, elevated non-canonical TGFβ signaling and increased dependency on autophagy/ER-stress response to maintain metabolic homeostasis. Notably, none of the inhibitors sensitizing BT20 cells with acquired resistance to dasatinib affect dasatinib resistance in parental cells reinforcing the notion that in resistant cells activation of the targeted pathways are activated in a SFK independent manner.

## MATERIALS AND METHODS

### Drug and compounds

3-Methyladenine (3-MA), Chloroquine (CQ), Rapamycin and Thapsigargin were purchased from Sigma-Aldrich. Dasatinib, buparsilib, (Selleck chemicals), salubrinal, SB431542, PI3-103 (Tocris Biosciences). BT20 cells were obtained from ATCC (Catalog No. 30-2003). BT20 cells with acquired resistance to dasatinib were obtained using a dose escalation protocol over a period of four months. BT20 cells were exposed to increasing concentrations of dasatinib in a stepwise fashion, starting from concentrations 100 times lower than their IC_50_ for two week [73]. In each cycle the dasatinib IC_50_ was assessed as described below. The selection process was repeated until the dasatinib IC_50_ concentration reached 1 μM. Parental cells were grown side by side to maintain equal passage numbers between parental and acquired resistant cells.

### Tissue culture and drug sensitivity

BT20 cells were maintained in culture at 37°C and 5% CO_2_ using Eagle's Minimum Essential Medium containing 10% FBS 10 U/mL penicillin (Wisent, QC, Canada) 10 μg/mL streptomycin(Wisent, QC, Canada). Cells (7500/well) were seeded in 200 μl in 96 well plates for 8-12 hours before treatment. Drug sensitivity was assessed as described previously using the sulphorodamine B (SRB) assay [74, 75]. Briefly, exponentially growing cells were seeded at low density in 96 well culture dishes to achieve an end point density within the linear range of the assay. After incubating overnight, cells were treated with vehicle or drugs alone or in combination. Five days post treatment cells were fixed with trichloroacetic acid, stained with SRB, and analyzed for percent growth on a 96 well plate reader. Efficacies of drug treatments were determined by calculating 50% inhibitory concentrations (IC_50_). Sensitization to dasatinib was assessed using interaction (I) values. I values were calculated by incubation with a dose-range of the dasatinib (0-5μM) in combination with vehicle (control) of a fixed non-toxic concentration of the drug of interest (IC_5_ concentration or less). According to the algorithm developed by Chou & Talay and Berenbaum [76, 77], when I<1, the interaction is synergistic, when I=1, there is additive or no interaction, and when I>1 there is an antagonistic interaction. I= [dasatinib IC_50_ in the presence of X/Dasatinib IC_50_ alone] + [concentration of X when combined with dasatinib/X IC_50_].

### Western blot analysis

Aliquots of treated cells as indicated were treated with whole-cell lysis buffer (10 mM Tris-HCl, 250 mM sodium chloride, 50 mM sodium fluoride, 0.5% Triton X-100, 0.1% sodium dodecyl sulfate, 10% glycerol, 1 complete pill of protease inhibitor mixture (Roche), 1 mM phenylmethysulfonyl fluoride, 100μM sodium orthovandate, 2mM iodoacetic acid, and 5 mM ZnCl_2_). Protein concentration was determined by Thermo Scientific Pierce BCA Protein Assay Kit following supplier stipulations. Equal amounts of protein for each sample were separated on a 4 to 12% Bis-Tris precast acrylamide gel (BioRad) and transferred to a nitrocellulose membrane. Western blots were probed with antibodies against the following antigens: Actin, Bip/GRP78, SMAD2/3, SMAD2/3 S423 and p27^KIP^ (Santa Cruz Biotechnology); 4E-BP1, 4E-BP1 Thr37/46, p70S6K Thr 389, ERK Thr202/204, FAKTyr861 and Y397, Src Tyr416 AMPK Thr172 (Cell Signaling); p21 (CIP1/WAF) (Neomarkers); LC3 I-II antibody (Novus Biologicals). The secondary antibodies were horseradish-peroxidase–conjugated anti-mouse (GE Healthcare), anti-rabbit (GE Healthcare) or anti-goat (Santa Cruz Biotechnology). Detection was performed by an enhanced chemiluminescent method (Immobilon Western, Millipore). Protein bands were quantified using ImagesJ 1.44p (Wayne Rasband, NIH) software and normalized to Actin.

### Apoptosis and cell cycle progression

Twenty four hours after treatment, floating and adherent cells were harvested, pelleted (1000g, 4°C for 5 min), resuspended in 1 ml of PBS, fixed by addition of 3 ml ice-cold 100% ethanol and incubated at 4°C for a minimum of four hours. The permeabilized cells were pelleted, washed with 2 ml of PBS, and stained in PBS containing 0.05 mg/ml propidium iodide (PI; Molecular Probes) and 0.2 mg/ml DNase-free RNaseA. The fraction of the population in each of the cell cycle (G0/G1, S and G2/M) was then calculated as a function of the DNA content, in comparison to vehicle treated cells. Apoptosis was detected by additional counterstaining using APC Annexin V (BD Biosciences) following the manufacturer instructions and described by us [14, 56]. All analyses were performed on FACS Calibur (Beckton Dickinson) flow cytometer equipped with Cellfit software.

### ROS detection

One million cells were plated in 6 well culture dishes and treated with Dasatinib IC_50_ for 12 hours. Cells were then incubated with 25 mg/ml 2′-7′-dichlorofluorescin diacetate (DCFH-DA- Invitrogen D-399) for 30 minutes at 37°C for the detection of intracellular peroxides (mainly H_2_O_2_). Cells were collected and washed in 1X dPBS. Fluorescence was measured by flow cytometry and analyzed using FCS Express v3.0.

### XBP1 splicing

XBP1 splicing was assessed by RT-PCR as previously described [78]. Briefly, cells were treated for 24 hours with vehicle, dasatinib or thapsigargin (positive control) as indicated. Cells were harvested, lysed and total RNA purified following the manufacturer's instructions (RNeasy, QIAGEN). RNA was reverse transcribed and amplified by RT-PCR according to the following protocol. 20 μg aliquots of total RNA were treated with M-MLV reverse transcriptase (Invitrogen) and then amplified with Ex-Taq polymerase (Invitrogen) using a pair of primers corresponding to nucleotides 412–431 (CCTTGTAGTTGAGAACCAGG) and 834–853 (GGGGCTTGGTATATATGTGG) of XBP1 cDNA. The resulting products were separated by electrophoresis using 1% agarose gels.

### Differential mRNA expression. RNA sequencing (RNA-Seq) and q-PCR

Differential gene expression between BT20 parental and acquired resistant cells were analyzed using RNA-Seq as described [30] by the analysis of 157,601 transcripts from Ensembl Hg19. Differential expression was assessed with adjusted FDR p-value < 0.1 and at least 2-fold expression change. The experiments were performed using the Illumina platform at Genome Quebec (www.genomequebec.com). Q-PCR was used to assess differential mRNA expression as described using specific primers for TGFβ2 (NM_009367) and normalized to GADPH (NM_008084) expression using a 7500 Fast Real Time PCR System from Applied Biosystems.

TGFB2 5′TCGACATGGATCAGTTTATGCG, 3′CCCTGGTACTGTTGTAGATGGA

GADPH 5′AGGTCGGTGTGAACGGATTTG, 3′TGTAGACCATGTAGTTGAGGTCA

### Statistical analysis

Statistical analysis was performed as indicated using Sigma Plot 13. Differences were considered significant with p<0.05 (www.sigmaplot.com).

## SUPPLEMENTARY MATERIALS FIGURES




