# Supplementary material for: Adaptive metabolic rewiring to chronic SFK inhibition
Source: Oncotarget. 2016 Mar 17;8(40):66758–68. doi: 10.18632/oncotarget.8146 (PMC5620134; doi:10.18632/oncotarget.8146)
Supplement: Supplementary file 1 [file oncotarget-08-66758-s001.pdf]

SUPPLEMENTARY MATERIALS

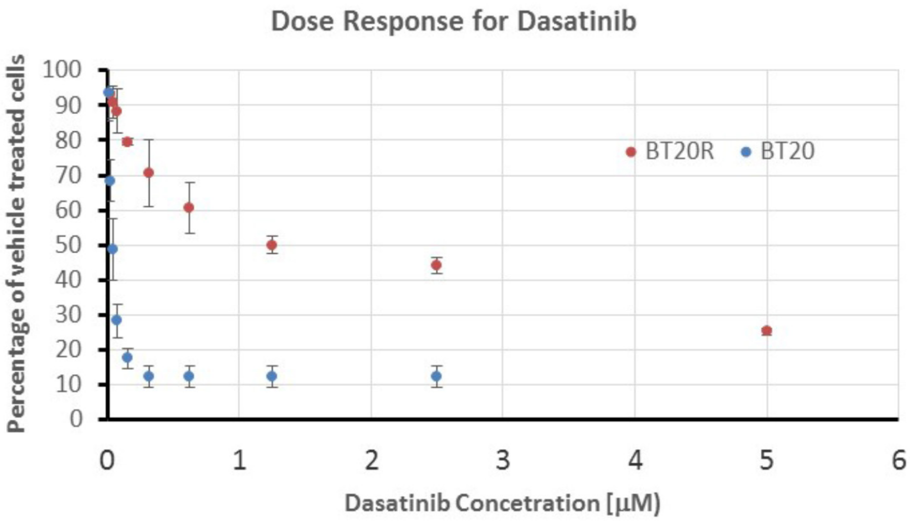

Supplementary Figure 1: Dose response curves used to obtain the IC50 values represented in FIGURE 1A.

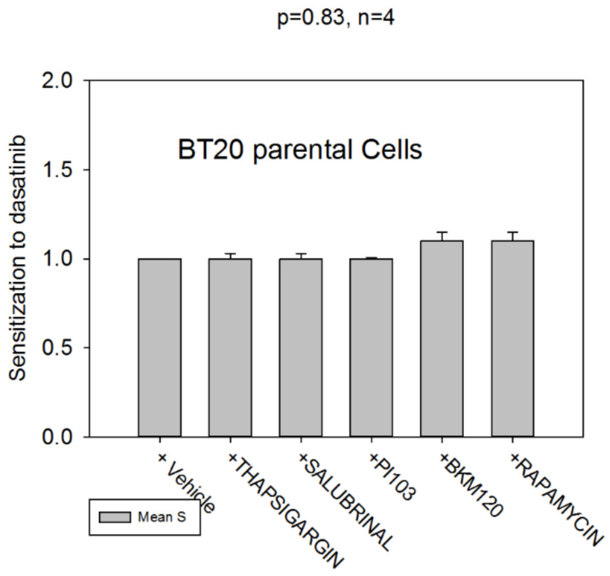

Supplementary Figure 2: Inhibitors used to assess sensitization in FIGURE 2E did not affect dasatinib sensitivity in BT20 parental cells (one way ANOVA, Holm-Sidak methods, n=4, p=0.83).
